# Sleep factors affecting mental health: mechanics and trigger factors

**DOI:** 10.3389/frsle.2025.1441521

**Published:** 2025-09-12

**Authors:** Kathy Sexton-Radek

**Affiliations:** ^1^Elmhurst University, Elmhurst, IL, United States; ^2^Hinsdale Hospital, Hinsdale, IL, United States

**Keywords:** sleep mental health, poor sleep quality, sleep, sleep health, insomnia

## Abstract

This article explores the concepts in sleep medicine associated with mental health symptoms. A brief overview of key sleep factors is stated, followed by a presentation of empirical research findings on how poor sleep influences mental health conditions. Some specific information on mental health conditions like insomnia, depression, and schizophrenia symptoms are provided. Tables and figures present common sleep conditions associated with mental health conditions. Additionally, a figure by the author proposes a pathway explaining the link between poor sleep quality and mental health conditions.

## Introduction

As a natural behavior, sleep drives the individual into a relaxed state of altered consciousness. This relaxed, altered state of consciousness–sleep rhythmically vacillates with an awake state on a 24-h cycle. From approximately the late teen years to the young adult years, the individual's sleep pattern of readiness for bedtime and a designated wake time are set (Sexton-Radek and Graci, 2023).

Sleep is characterized as non-rapid eye movement (nREM) sleep with decreasing varying degrees of awareness corresponding to increased sleep state (i.e., nREM1, nREM2, nREM3/4) from 1 to 4 (Borbely, 1982). Rapid eye movements from left to right, along with low muscle tone, temperature dysregulation, and brain activity, such as wake activity, characterize rapid eye movement (REM) sleep (Anderson and Bradley, 2013; Baldini et al., 2019; Freeman et al., 2020). However, some individuals with mental health symptomology experience disturbed sleep. The typical night of sleep is four cycles of non-REM stage 1 sleep, non-REM stage 2 sleep, non-REM stage 3/4 sleep, and then REM sleep of ~90-min intervals during sleep sequences. Borbély's model proposes that sleep is determined by two factors: sleep homeostat balance between sleep need and sleep and an individual's circadian rhythm. With this, behavioral factors, such as stress and sad mood, can influence these physiological factors (Borbely, 1982; Hale et al., 2020).

## Good sleep is good health

**Figure 1:**
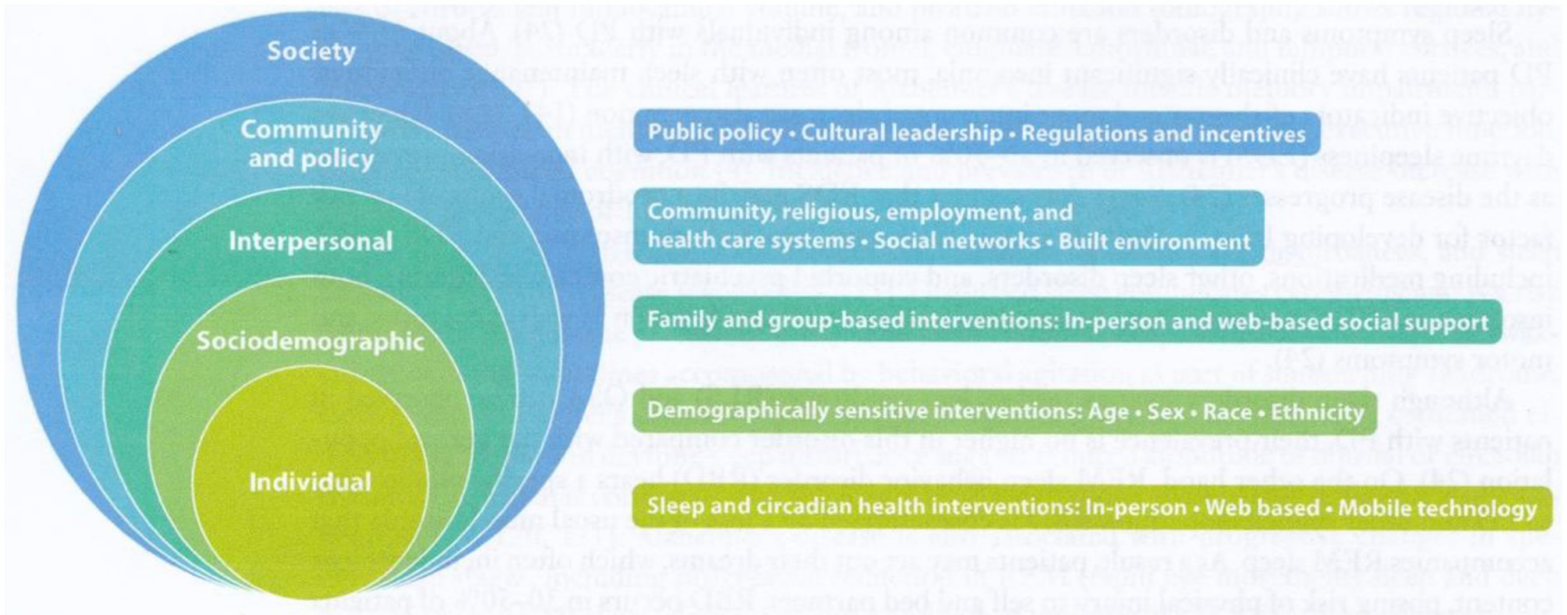
The socioecological model of sleep and circadian health with corresponding multilevel intervention stratergies. Source: Grandner (2019).

Buysse (2014) commented that sleep health indicates how well an individual or population is doing. The balance that exists between sleep states and the wake state is similar to the internal homeostat that generates sleep, called our sleep–wake cycle. It is anticipated that total sleep time, on average, for adults is 6.5–7.5 h, with 15–30 min for falling asleep. Variations from this rhythm are sleep disturbances and, when intensified, can become a sleep disorder. For most, the timing of sleep is typically at night, and with the homeostat, the sleepiness builds, taking place some 14–16 h after wakefulness. Buysse's (2014) sleep factors concept is illustrated in Figure 1. Sleep factors from environmental influences at the society and community levels, as well as individual factors, are considered influential in an individual's sleep quality. At the individual level, genetic components of cellular processes, along with systemic processes of immune, endocrine, and sympathetic nervous system processing, may be protective or dysfunctional, such as inflammation (Foster et al., 2013). A dysfunction, such as inflammation, at the endocrine level can lead to poor health outcomes. Hale et al. (2020) underscore the value of considering additional variables related to sleep health in terms of sociocultural factors. Furthermore, multiple contextual variables, such as family, school, workplaces, media, and policy, influence sleep patterns (Lee et al., 2023).

## Behavior influence on sleep determinants

**Figure 2:**
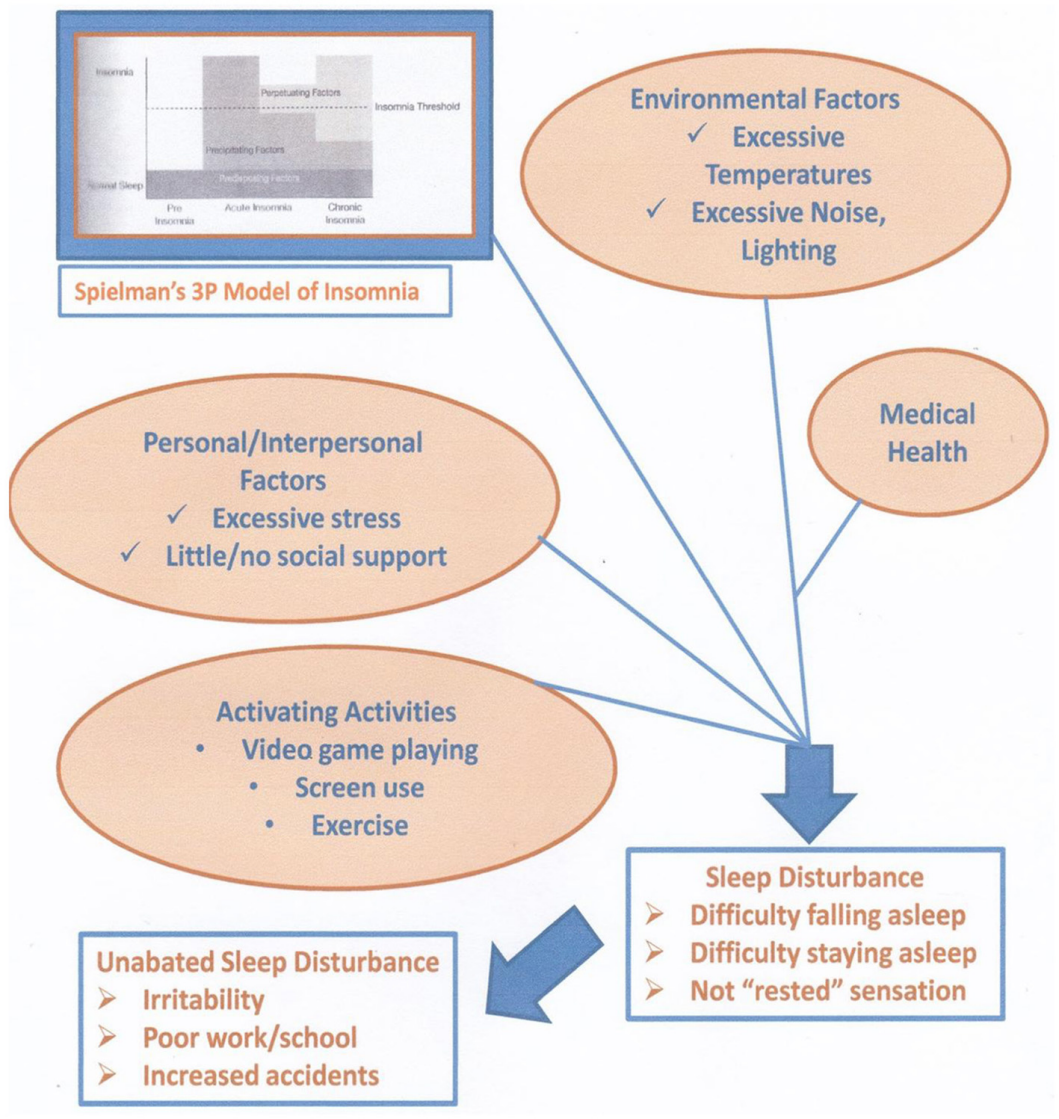
Proposed mechanism to sleep disturbance. The Spielman model of insomnia - 3P, predisposing, precipitating, and perpetuating (adapted with permission from Spielman et al. 1987, A Behavioral Perspective on Insomnia Treatment - ScienceDirect).

Internal or external provocation, such as the behavioral states of anxiety or symptoms of medical conditions, can disturb sleep. Seng et al. (2016) reported that one in four adults in the United States meets the criteria for a sleep disorder. Estimates using standard measures (e.g., Insomnia Severity Index, Berlin Questionnaire) have led to identifying insomnia as an unmet need (Seng et al., 2016). The researchers also concluded that their screening results were likely to underestimate urban, ethnic populations. Figure 2 illustrates the influential role of sleep health to an individual/society.

Investigations into sleep quality and cognitive behavior therapy (CBT) treatment of patients with insomnia have identified gains in symptom reduction once the insomnia symptoms are reduced (Sexton-Radek and Graci, 2023). Figure 2 illustrates the factors related to the onset of insomnia symptomology. Dr. Spielman provided the foundational theory that looks at the predisposing, precipitating, and perpetuating facts (i.e., the “three Ps”) that disturb an individual's sleep (Ellis et al., 2021). Sleep disturbance onset of signs and symptoms of the insomnia pathway can be examined in terms of these factors at an individual level for the sleeper. Increased treatment and potential threats to mental health may occur from an imbalance of personal/intrapersonal relationships, little or no support, and excessive stress (Sexton-Radek and Graci, 2023; Wittman et al., 2006). Furthermore, extremes of environmental factors and undetected/untreated medical conditions may sufficiently strain an individual as sleep becomes disturbed (Talbot et al., 2014). With this, life choices of variable sleep (e.g., changing bedtimes and waketimes and napping) and excessive screen use may contribute to poor sleep quality. Mental health changes when on becomes distressed and leads to a non-rested state (Babson et al., 2010; Waters et al., 2018). This “not rested” state of sleep deprivation affects mental health, with cognitive deterioration of impaired/erratic attention and concentration levels, impulsive emotional states, and negative emotional states (Fernandez-Mendoza et al., 2015; Gangwisch et al., 2010; Gunderson et al., 2023; Hogenkamp et al., 2013; Sexton-Radek and Graci, 2023). Mental health states lead to heightened arousal, which is contrary to the relaxed state essential for falling asleep (Sexton-Radek and Graci, 2023). The Assessment of Sleep and Treatment of Sleep sections underscore the importance of treating sleep disturbance as a countermeasure to developing or worsening emotion dysregulation and cognitive function deficits (Clement-Carbonell et al., 2021; Drake et al., 2006; Sexton-Radek and Graci, 2023). The development of poor mental health symptoms, such as depression and anxiety, worsens poor sleep health, and poor sleep increases a person's vulnerability to increased mental health and possible triggers to disorders' onset (Babson et al., 2010; Loddo et al., 2019). Effective treatments of poor sleep quality with CBT–insomnia (CBTi) interventions also reduce mental health symptomology (Babson et al., 2010; Cho et al., 2015; Cappaccio et al., 2010; Bonnet et al., 1985; Drake et al., 2006; Goodwin and Marusic, 2008; Sexton-Radek and Graci, 2023).

## Assessment of sleep

A thorough evaluation of a patient's symptoms and symptom pattern provides clinicians with a means of conceptualizing a patient's treatment pathway. In the sleep medicine field, specialized measures are used in addition to a standard clinical interview, common personality measures such as the Minnesota Multiphasic Personality Inventory (MMPI) and the Millon Clinical Personality Inventory, and a sleep diary are used. The specific measures of the Pittsburgh Sleep Quality Index (PSQI) and the Epworth Sleepiness Scale (ESS) provide the clinician with specific sleep patterns, sleep disturbance, and sleep quality information necessary for diagnosis and treatment planning. The PSQI has 19 questions focusing on individual reports using a Likert rating scale. The total score varies from 0 to 21, with higher scores indicative of sleep disturbance. A PSQI score >5 is regarded as clinically relevant to a sleep disturbance. The ESS is an 8-item listing of situations that the individual is requested to rate in terms of the likelihood of feeling sleepy (e.g., stopped at a red light while driving). A sleep diary is frequently used to assess sleep. The sleep diary requires a daily recording of sleep onset, number of wake-ups during the night, and the timing of wake-ups, Whether the patient tracks with their Apple Watch or Fitbit or using the standard paper-and-pencil farm, the following are noted daily: bedtime, number of minutes until sleep, wake-up time, time out of bed, number of wakings, time and length of wake-ups, napping time, and assessment. By using a sleep diary to inquire about the features of the patient's night of sleep, sleep efficiency can be calculated by subtracting the number of minutes asleep from the total number of minutes in bed. A sleep efficiency of 85% is adequate (Sexton-Radek and Graci, 2023). From here, the sleep clinician may select specific measures according to the presenting problems, such as attitude toward sleep, sleep style—morning or evening person, and presence of dysfunction varies about sleep (Benham, 2006; Foster, 2020; Natale et al., 2006; Poon et al., 2024). If a patient case presents a challenge about the dimension of their sleep despite the measures, a more precise measure of sleep using an Actiwatch (wrist device correlated to polysomnogram) that a patient wears on the wrist, and the accelerometer device within the Actiwatch is synchronized to software (Fekedulegn et al., 2020). In this manner, sleep onset, sleep offset, and the amount of movement are accurately recorded. An all-night polysomnogram is used for cases in which the sleep clinician believes may have symptoms of restless leg syndrome (RLS), obstructive sleep apnea, parasomnia, or other sleep disorders. Other standard measures used by sleep specialists to assess sleep include a structured interview, the Beck Depression Inventory, and the MMPI.

## Treatment of sleep (sleep disturbance and insomnia)

CBTi treats sleep disturbances and provides a means of ongoing assessment and diagnosis. This treatment has a cognitive behavior therapeutic orientation; thus, the sleep specialist collaborates with the patient to understand and change the sleep pattern to a healthier version. Behavioral techniques, such as stimulus control (i.e., assignment of a task to distract the patient from the potentially controlling aspects of a circumstance, such as wakefulness), and sleep restriction, a technique to use the optimal amount of sleep, are primarily emphasized in CBTi. The secondary focus—sleep hygiene, sleep environment, and learning to relax—is taught therapeutically by the sleep specialist.

CBTi can be conducted concurrently with other therapies the patient is already receiving (i.e., individual and/or group). It may be the case that the elements of the psychiatric symptoms a patient is experiencing, such as anxiety, may be related to or influenced by their sleep disturbance. On a general level, poor sleep quality and sleep deprivation influence a person's functioning. Correlational studies have identified that sleep deprivation worsens with symptom severity. In CBTi, another focus is to examine the person's 24 hour pattern-to include their wake day events that may be influential to their nighttime sleep. Thus, not only feeling sleep deprived but also factors such as sedentary lifestyle, poor nutrition, and overuse of caffeine and stimulants, such as nicotine, worsen sleep quality (Ramar et al., 2021).

Treating sleep disturbance, educating about sleep health, and treating insomnia are important, influential factors in the successful psychiatric care of patients (Seth et al., 2005). At the superficial level, CBTi instruction on self-care for sleep health is a positive element; the therapeutic encounter with a sleep specialist is impactful, and the outcome of adequate sleep facilitates the patient's work toward more positive mental health. In addition to psychotherapy and CBTi, the sleep specialist may recommend a medication evaluation to determine the need for pharmaceutical treatment (Sexton-Radek and Graci, 2023). Hypnosis and melatonin-inducing medication are considered for complaints of insomnia. Some antidepressant medications are used in dosages and dosings that vary from traditional depression treatment regimens as they have sedating effects (Medic et al., 2017; Meerlo et al., 2015). With some less prevalent sleep disorders, such as hypersomnia, and narcolepsy, amphetamine formulations are considered.

Based on precise research work, the use of a bright light lamp provides timed exposure at periods crucial to the visual pathway, leading to success in cases of seasonal affective disorder (Faulkea et al., 2019). Some cases of sleep maintenance insomnia, where the patient's sleep difficulty is staying asleep, also benefit from bright light therapy (Li et al., 2024).

## Psychiatric conditions commonly associated with sleep

Psychiatric diagnoses of depression, posttraumatic stress disorder (PTSD), generalized anxiety disorder, and schizophrenia involve a component of stress. The stress of the experiences of the diagnoses may be increased with the vulnerability of poor sleep (Crouse et al., 2012; Krystal, 2012; vanMill et al., 2014). Loneliness and social isolation escalate stress symptoms and worsen the social isolation symptoms common in psychiatric disorders. As a result of the increased emotionality associated with psychiatric conditions, sufficiently relaxing for sleep may be difficult. Disrupted sleep provokes the sleeper to frequent awakenings. With significant emotional challenges such as flashbacks to the trauma in PTSD the risk of disturbed sleep increases. Tables 1, 2 list some clinical features of sleep disorders of arousals that may be experienced by patients with extremely disruptive emotional symptomology (Sun et al., 2022; Uhde et al., 2009).

**Table 1:** Clinical features of disorders of arousal.

**Patient variable**	**Confusional arousal**	**Sleepwalking**	**Night terrors**
Age	2–10 years	4–12	18 months−10 years
Onset	First third of night	First third of night	First third of night
Agitation	Mild	No/poor	Marked
Medium	Medium	Mild	Marked
Motor activity	Low	Complex	Rarely complex
Behavior	Whimpering, some articulation, sitting up in bed, inconsolable	Screaming, agitation, flushed face, sweating, inconsolable	Walking around, quiet or agitated, unresponsive to verbal commands
Amnesia	Yes	Yes	Yes
Threshold of arousal	High	High	High

Source: Loddo et al. (2019).

**Table 2:** Classification of parasomnias based on *International Classification of Sleep Disorders*, Third Edition.

**nREM-related parasomnias**	**REM-related parasomnias**	**Other parasomnias**
Confusional arousals	REM sleep behavior disorder	Exploding head syndrome
Sleepwalking	Recurrent isolated sleep paralysis	Sleep-related hallucinations
Sleep terrors Sleep-related eating disorder	Nightmare disorder	Sleep enuresis

Source: American Academy of Sleep Medicine (2014). nREM, non-rapid eye movement; REM, rapid eye movement.

## Neurobehavioral markers related to sleep

Andrillon et al. (2016) examined sensory activation using an evoked potential metric while the participant classified words with hand gestures. This procedure allowed neural complexity to be measured. This dependent measure varies across REM and nREM stages of sleep. As participants fell asleep, cortical functioning changed from nREM1 to nREM2 sleep and from nREM2 to nREM3/4 sleep to REM three-fourths sleep to REM sleep. Table 1 reflects a series of clinical features of cerebral changes in activity by sleep state. Wakefulness and nREM states have the highest rates of arousal. Furthermore, Andrillon et al. (2016) identified distinct responses for anticipating motor responses, with the decreased ability for adequate motor responses in the deeper stages of sleep.

Findings from neuroscience sleep research have identified that healthy sleep can lead to a balanced mood and brain metabolites being adequately cleared. Sleep research has documented the clearance of brain metabolites during sleep (Watson et al., 2006). In both clinical and neuroscientific studies, insomnia has been common in investigations of mental health and sleep. Usually, short-sleep-length variables, particularly 6 hours or shorter, not only correspond to self-reported “poor sleep” but are also considered to be associated with trigger factors and worsening sleep in mental health conditions. Some studies of sleep duration have examined fragmented sleep-−1 or 2 h of sleep and then awake, followed by a sleep interval and a wake interval (Nickolmann et al., 2007; Winsper and Tang, 2014; Wittman et al., 2006). Abbreviated sleep and abbreviated uninterrupted sleep make the sleeper vulnerable to changes in their mood (Pieroni et al., 2023; Soehner et al., 2013). The absence of adequate sleep results in negative (e.g., easy to frustrate, irritable, sad, and reactive) mood states (Pankowska et al., 2020).

The loss of prefrontal cortex control resulting from sleep deprivation contributes to a person losing control over emotions. Given that a negative mood state reflects this lack of cortical control to filter an irritable mood state (McCoy et al., 2011; Pigeon et al., 2017; Sateia, 2011). This cortex dysregulation results in the emotion dysregulations of depression, anxiety, and aggression (Hobson and Friston, 2014; Lipinska et al., 2015). The decreased concentration, attention, and ability to react, along with the emergence of emotions, results in emotions experienced with little self-control and impaired reading of and reacting to the context of an emotion (Ellis et al., 2021; Nickolmann et al., 2007). Figure 2 depicts the common disruptions to relaxation, essential to falling asleep, at presleep—a contrast of two events from the sleep environment to many commonly reported disruptions.

Nationwide survey results have identified an inverse relationship between psychiatric comorbidities and healthy sleep (Soehner et al., 2013). Thus, not only feeling sleep deprived but also factors such as sedentary lifestyle, poor nutrition, and overuse of caffeine and stimulants, such as nicotine, worsen sleep quality. The following personal factors were identified as present in the participant groups: high levels of stress, social isolation, loneliness, and social cohesion. Additionally, researchers have reported community factors, including excessive noise and light pollutants, extremes of temperature, and personal safety threats due to chaotic traffic, crime exposure, and poverty (Andrillon et al., 2016; Buysse, 2014; Cho et al., 2015; Meerlo et al., 2015; Seng et al., 2016).

When treating sleep disturbance, education about sleep health and treatment for insomnia is an important, influential factor in the successful psychiatric care of patients. At the superficial level, CBTi instruction on self-care for sleep health is a positive element, the therapeutic encounter with a sleep specialist is impactful, and the outcome of adequate sleep facilitates the patient's work toward more positive mental health. In addition to psychotherapy and CBTi, sleep specialists may recommend a medication evaluation to determine the need for pharmaceutical treatment. Hypnosis and melatonin-inducing medication are considered for complaints of insomnia. Some antidepressant medications are used in varying dosages and dosing for traditional depression treatment regimes. With some infrequent sleep disorders of hypersomnia and narcolepsy, amphetamine formulations are considered.

## Parasomnia, narcolepsy, and RLS

Of relevance to psychiatric conditions are the symptom patterns of parasomnia, narcolepsy, and restless legs (Sexton-Radek and Graci, 2023). Parasomnias manifest as events alongside sleep and are characterized as REM-related parasomnias and non-REM-related parasomnias. The REM-related parasomnias are REM behavior disorder, recurrent isolated sleep paralysis, and nightmare disorder. Non-REM-related disorders include confusional arousal, sleepwalking, sleep terrors, and sleep-related eating disorders. Some parasomnia variants are exploding head syndrome, sleep-related hallucinations, and sleep enuresis (Loddo et al., 2019; Picchetti and Winkleman, 2005; Poon et al., 2024).

The features of parasomnia conditions are assessed by structured clinical interview, specialty scales, and, if needed, an all-night polysomnogram. Considering some similarity in symptoms of confusion, fears, partial awakenings, and psychiatric symptomology—particularly anxiety and depression disorders—is possible. Here again, intensive, specialty measures and an all-night sleep study could discern the symptoms associated with a psychiatric diagnosis or a sleep condition.

Hypersomnia and narcolepsy are assessed using measures and a sleep study. Treatments for symptoms of sleeplessness and sleep attacks characterized by these conditions are effective. The disrupted sleep patterns of narcolepsy include disturbing dreamlike states at the start and end of sleep. This phenomenon, along with sleep attacks, has been identified as difficult to diagnose by non-sleep specialists, resulting in some patients receiving misdiagnoses of schizophrenia, multiple sclerosis, and other psychiatric diagnoses. Table 2 displays the clinical features of disorders of arousal (Ju et al., 2013; Kamath et al., 2015; Sexton-Radek and Graci, 2023; Yaffe et al., 2011).

Occurring during presleep, RLS is characterized by the urge to move one's legs. If the leg and/or other limb movements continue during sleep and they meet the criteria patterns, the person is diagnosed with periodic limb disorder. RLS is diagnosed by interview. RLS is treated palliatively with exercise and leg stretches, review and upgrade of nutrition and hydration, and stimulus control behavioral interventions. If severe, pharmacological treatments are provided for patients. Tables 1, 2 list the clinical features of arousal from sleep.

## Sleep presentation and psychiatric disorders

Sleep complaints are common in both the general population and those with psychiatric disorders. Sleep disturbances represent a risk factor that can complicate the condition and jeopardize treatment and treatment outcome. Sleep disturbance complaints often predate symptoms of depression, PTSD, and anxiety disorders.

The causality of sleep factors to psychiatric symptoms has been speculated with equivocal research support (Ramos et al., 2023; Sexton-Radek and Graci, 2023; Thakkar et al., 2015). While common neurotransmitters participate in both conditions (i.e., serotonin, epinephrine, and dopamine), the timing and amounts vary by condition, thus rendering a common pathology unlikely (Spira et al., 2013; Tasali et al., 2008; Troyel et al., 2012). For poor sleep quality with frequent wake-ups, the difficulty may increase the patient's vulnerability to other neurochemical changes associated with psychiatric disorders.

Patients diagnosed with attention-deficit/hyperactivity disorder complain of their sleep. Sleep is often disturbed in terms of insomnia at sleep onset or sleep maintenance insomnia. In autism spectrum disorder, patients also commonly complain about the difficulty of falling asleep. Here again, a CBT intervention to resolve poor sleep may address and reduce some acute stress disorder symptoms (Talbot et al., 2014).

Anxiety disorders, such as panic disorder, often co-occur with insomnia. Relatedly, obsessive-compulsive disorder is associated with poor sleep secondarily, given time commitments to complete requisite compulsive behaviors. In PTSD, the precipitating event, unless resolved, continues to evoke restlessness and discomfort sufficient to counter the necessary relaxation to fall asleep (Picchetti and Winkleman, 2005; Sexton-Radek and Graci, 2023; Vanaelst et al., 2012; Wilkins and Moroz, 2009). Parasomnia and insomnia are associated with PTSD diagnoses. The few empirical studies in this area using polysomnogram (PSG) measures have identified increased sleep latency and decreased REM onset latency in an anxiety disorder population (Sexton-Radek and Graci, 2023).

Depression and bipolar disorder, mood disorders, are typically associated with amounts of sleep disruption and sleep stages. Sleep architecture is disrupted, and in cases of depression, the time to REM is shortened. With schizophrenia, hallmark symptoms of psychosis, thought disorder, and behavior and perceptual disorders often co-occur with complaints of poor sleep. Several studies have reported an increased sleep onset latency. Patients with schizophrenia and insomnia diagnoses commit suicide at higher rates than those with a schizophrenia diagnosis alone. Stage 2 abnormalities, as well as circadian abnormalities are common in the sleep of patients diagnosed with Schizophrenia (Winsper and Tang, 2014).

## Research on disturbed sleep

Sleep deprivation studies provide a window into the effects of poor sleep on behavior (Buysse et al., 1992; Dombrowski et al., 2008). The common finding is familiar: increased irritability. Research findings have categorized irritability from patient ratings and by observing participants who self-reported disturbed sleep (Babson et al., 2010). Additionally, irritability subsides with improved sleep. Sleep deprivation increases one's perception of pain. Pain sensitivity is increased in patients with fragmented sleep, such as with some medical conditions (Jennings et al., 2007). In instances of transitional pain conditions secondary to injury or fall as distinguished from chronic pain fragmented sleep coexists (Sexton-Radek and Graci, 2023). Diminished recall and long-term memory occur with sleep deprivation (John et al., 2005; Milner et al., 2009; Pigeon et al., 2017). Recent neurobehavioral studies have identified the role of erratic and reduced prefrontal functioning, an organizer of memory, in sleep-deprived patients. Alterations in autonomic activity occur in depression and anxiety. The hypothalamus–pituitary–adrenal axis is activated by actual or perceived stress, which, in turn, contributes to erratic neurotransmitters as well as a deficit in their number. This emotional stress response causes increased activity, resulting in dysregular brain activity and sustained stress responds (Andrillon et al., 2016; Ju et al., 2013; McCoy et al., 2011; Oh et al., 2019; Olcese et al., 2018; Pankowska et al., 2020; Ramos et al., 2023).

## Emotional trigger factors of sleep disturbance

The Spielman model of insomnia provided a theoretical means to determine the formation of sleep disturbance. For example, a patient who may be a “light” sleeper (i.e., easily woken from sleep) undergoes a medical procedure, and while in the hospital, postoperative checks for blood pressure and the healing level of the incision during the night may interrupt their sleep. When the patient is transitioned to home, the interruptions in sleep during recovery may continue and become perpetual or sustaining factors of the sleep.

Research studies have identified associations between extremes of temperature, sound, light, and sleep disturbances (Boland et al., 2015; Faulkea et al., 2019; Ramos et al., 2023; Sexton-Radek and Graci, 2023). Activating events, such as exercise, increase blood pressure and adrenalin, contrary to the essential relaxation needed for sleep. Excessive screen use such as viewing social media is visually and cognitively activating to the point of also blocking the relaxation necessary for sleep. Figure 2 depicts the interrelationship of triggering factors that lead to poor sleep. With a relationship between poor sleep health and reduced mental health, focusing on these factors in patient care becomes essential (Dombrowski et al., 2008; Goodwin and Marusic, 2008; Hogenkamp et al., 2013; Kamath et al., 2015; Medic et al., 2017; Pigeon et al., 2017; Sexton-Radek and Graci, 2023).

## Summary and implications

Sleep health is essential to mental health. Disturbances in sleep in terms of shortened length, disruptions, and waking from sleep have a primary effect on the sleeper's daytime sleepiness and dissatisfaction with sleep. However, within the context of the serious issue of mental health, poor sleep increases an individual's vulnerability to developing poor mental health as well. Presented were studies and themes that identified the behavioral, emotional, and cognitive changes in a sleep-deprived individual that impact their stress response—in that it becomes sustained. Thus, in patients with mental health challenges, worsened states are typically measured. Assessing and treating the common sleep disturbance of insomnia were presented in terms of empirically validated measures and treatments. A resounding message of poor sleep's influence on mental health, evidenced by several correlational studies, leads to the recommendation of individualizing treatment and addressing the preemptive sleep disturbance first or concurrently to reduce discomfort, development, and worsened symptoms in a patient with mental health conditions.

## Resources for sleep and mental health issues

American Academy of Sleep Medicine: http://www.aasm.org
National Sleep Foundation: http://www.thensf.org
National Institutes of Health (NIH): http://www.nih.gov
World Sleep Society: http://www.worldsleepsociety.org
